# The rat model of COPD skeletal muscle dysfunction induced by progressive cigarette smoke exposure: a pilot study

**DOI:** 10.1186/s12890-020-1109-y

**Published:** 2020-03-23

**Authors:** Jianqing Su, Jian Li, Yufan Lu, Ning Li, Peijun Li, Zhengrong Wang, Weibing Wu, Xiaodan Liu

**Affiliations:** 1 Department of Sports Medicine, Shanghai University of Sport, Shanghai, 200438 China; 2 School of Rehabilitation Science, Shanghai University of Traditional Chinese Medicine, Shanghai, 201203 China; 3 Institute of Rehabilitation Medicine, Shanghai Academy of Traditional Chinese Medicine, Shanghai, 201203 China

**Keywords:** Chronic obstructive pulmonary disease, Skeletal muscle dysfunction, Modeling methods,a pilot study

## Abstract

**Background:**

Chronic obstructive pulmonary disease (COPD) skeletal muscle dysfunction is a prevalent malady that significantly affects patients’ prognosis and quality of life. Although the study of this disease has attracted considerable attention, a definite animal model is yet to be established. This study investigates whether smoke exposure could lead to the development of a COPD skeletal muscle dysfunction model in rats.

**Methods:**

Sprague Dawley rats were randomly divided into model (MG, *n* = 8) and control groups (CG, *n* = 6). The MG was exposed to cigarette smoke for 16 weeks while the CG was not. The body weight and forelimb grip strength of rats were monitored monthly. The pulmonary function and the strength of tibialis anterior muscle were assessed in vitro and compared after establishing the model. The histological changes in lung and gastrocnemius muscles were observed. The expressions of interleukin (IL)-6, IL-8, and tumor necrosis factor (TNF)-α were detected by ELISA, while the expressions of Atrogin-1 and MuRF1 in the gastrocnemius muscle were determined by Western blotting.

**Results:**

Smoke exposure slowly increases the body weight and forelimb grip strength of MG rats, compared to CG rats. However, it significantly decreases the pulmonary ventilation function and the skeletal muscle contractility of the MG in vitro. Histologically, the lung tissues of MG show typical pathological manifestations of emphysema, while the skeletal muscles present muscular atrophy. The expressions of IL-6, IL-8, and TNF-α in MG rats are significantly higher than those measured in CG rats. Increased levels of Atrogin-1 and MuRF1 were also detected in the gastrocnemius muscle tissue of MG.

**Conclusion:**

Progressive smoking exposure decreases the contractile function of skeletal muscles, leading to muscular atrophy. It also increases the expressions of inflammatory and muscle protein degradation factors in COPD rats. This indicates that smoke exposure could be used to establish a COPD skeletal muscle dysfunction model in rats.

## Background

Chronic obstructive pulmonary disease (COPD), a malady induced by air pollution and aging [1–3], is the third leading cause of death in the world [4, 5]. To enhance the quality of life of COPD patients, it is necessary to reduce the disease’s symptoms and side effects, including skeletal muscle dysfunction. This condition implicates reduced strength or endurance of skeletal muscles, especially the lower limb muscles represented by quadriceps femoris and gastrocnemius, and it occurs in more than 90% of COPD patients [6]. Ultimately, the muscular atrophy limits the physical activity of patients, thereby affecting their quality of life [7]. Moreover, it has been recently shown that skeletal muscle dysfunction is directly related to the mortality rate of COPD. In fact, for patients diagnosed with moderate to severe pulmonary dysfunction, the strength of the quadriceps femoris muscle is considered to be a better indicator of COPD mortality than the forced expiratory volume in the first second (FEV1) [8]. Therefore, in order to develop a rational and effective treatment of COPD, it is essential to acquire a profound understanding of the biological mechanisms leading to the instigation of skeletal muscle dysfunction.

Animal models play an important role in basic physiological research. However, despite the numerous studies conducted on COPD skeletal muscle dysfunction, a clearly defined animal model of this disease is yet to be established. Recently, it has been reported that long-term cigarette smoke exposure (CSE) induces muscle loss, such as pathological atrophy of quadriceps femoris, in COPD mice [9]. In fact, after 24 weeks of CSE, the growth of skeletal muscles in COPD mice was found to be significantly inhibited, and skeletal muscle atrophy was observed [10]. Another study showed that 8, 16, 24 and 32 weeks of CSE leads to decreased motor ability and weakened skeletal muscles in mice [11]. However, despite the reduced motor ability, the muscle and mitochondrial functions in COPD mice remain intact after 20 weeks of exposure to cigarette smoke [12]. Based on these results, it may be concluded that CSE inhibits the muscle function in model animals, thereby causing skeletal muscle dysfunction. This indicates that CSE can be used to establish a COPD skeletal muscle dysfunction model in animals. It should be noted though that the available studies supporting this conclusion are scarce, and that a scientific evaluation system still needs to be developed in order to explore animal models of COPD skeletal muscle dysfunction.

COPD skeletal muscle dysfunction is manifested in the form of functional and structural changes. Patients diagnosed with this condition exhibit limited overall motor ability or decreased contractility of individual muscles. Concurrently, the skeletal muscles of these patients present signs of atrophy and fibrosis. Changes in muscle fiber types have also been observed in COPD patients. Studies of COPD skeletal muscle dysfunction suggest that this disease develops via a variety of molecular mechanisms, including cell autophagy, oxidative stress, and systemic inflammation [13]. Moreover, imbalances in myofibrillar protein synthesis and degradation play an important role in muscular dysfunction. In fact, the ubiquitin-proteasome system (UPS) responsible for degrading proteins in muscle cells is considered to be one of the specific markers of skeletal muscle dysfunction [14]. In particular, the MuRF1 and Atrogin-1, E3 ligases are implicated in recognizing degraded proteins, as shown by animal experiments and clinical studies [15, 16]. Thus, their expressions directly reflect the level of muscle wasting in COPD patients. This indicates that changes in muscle function, structure, and related molecular levels may be used as criteria for the evaluation of skeletal muscle dysfunction in COPD patients.

To confirm whether or not the existing long-term CSE animal models may be used as models of skeletal muscle dysfunction, we assessed the effect of 16-week incremental, systemic smoke exposure in inducing skeletal muscle dysfunction in COPD rats. The experiments were performed based on the available schemes of COPD model development. Skeletal muscle contractility, histopathological observations, and changes in muscle protein degradation levels were used to evaluate the success of the prepared model. The results reported herein may be used to develop treatments of COPD skeletal muscle dysfunction in future studies.

## Methods

### Animals and management

Fourteen healthy male Sprague-Dawley rats, aged 2 months and weighing an average of 200 ± 20 g, were purchased from Witong-lihua Laboratory Animal Technology Company (production license: Zhejiang SCXK2018–0001). The animals were housed in 465 × 328 × 197 mm^3^ cages in which they acclimated to a 12-h circadian rhythm. They were also permitted ad libitum access to food and water in an environment maintained at 21 ± 2 °C and 60% ± 10% humidity. The study was conducted in strict accordance with the ethical codes, and all measures were taken to alleviate the pain of animals during the course of experiments. All research protocols developed herein had been approved by the Institutional Animal Care and Use Committee of Shanghai University of Sport (No.2018026) before implementation.

### Establishment of the COPD skeletal muscle dysfunction model

After 1 week of adaptive feeding, the fourteen rats were randomly divided into model (MG, *n* = 8 rats) and control (CG, *n* = 6 rats) groups. To reduce subjective bias, the random and blind methods were applied during classification. The rats were labeled based on their body weights, and they were assigned random numbers using Microsoft Excel. Rats with single-digit numbers were included in MG, while those with double-digit numbers were considered to be CG rats. The entire process of random classification was conducted by a third person who did not participate in the experiment, so as to ensure the applicability of the blind criterion. As a pilot study, we selected theoretically significant minimums for the number of rats in each group.

**Fig. 1:**
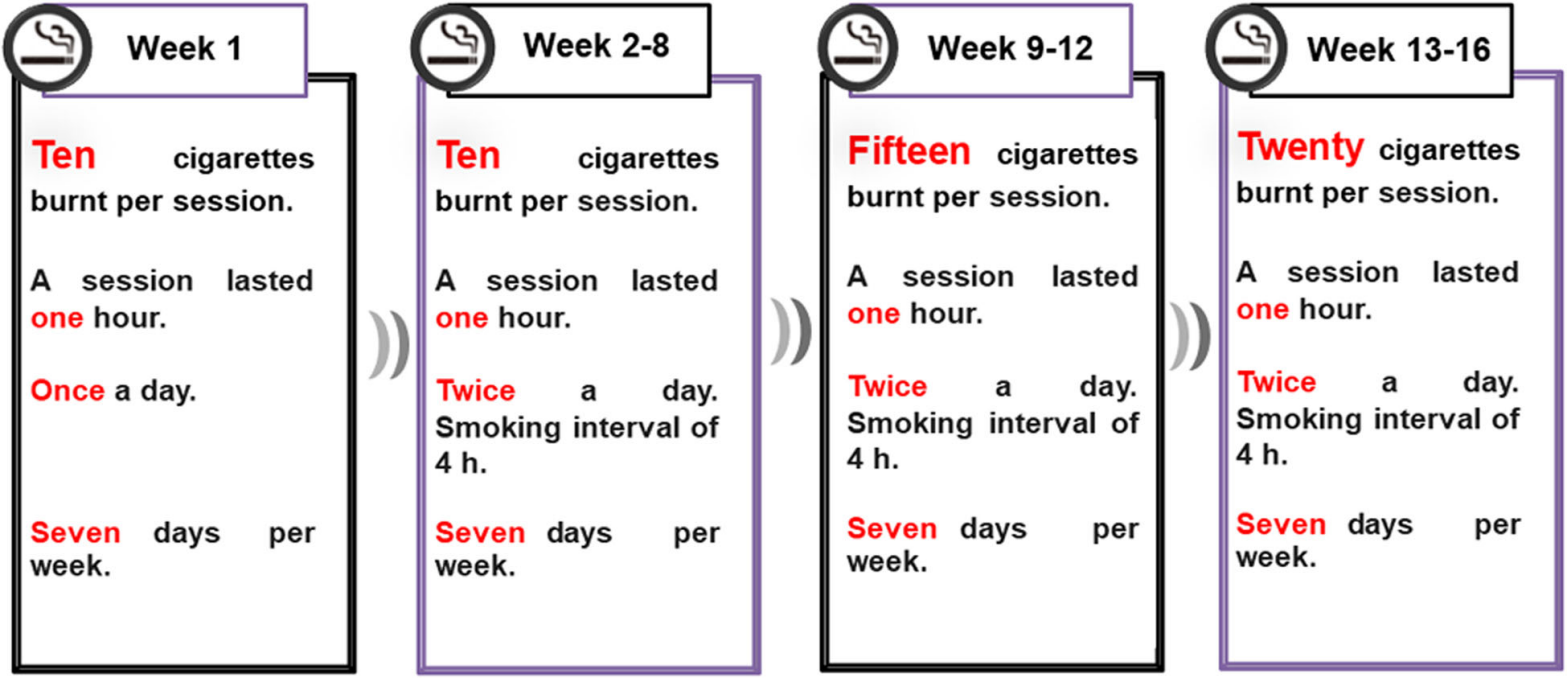
Progressive cigarette smoke exposure scheme

The model rats were subjected to cigarette smoke, while those in CG were exposed to fresh air. The process of incremental systemic CSE was started in July 2018, and it ended in October of the same year. The CSE scheme adopted in this study was taken from a previous publication [17]. Briefly, the eight rats in MG were placed in a passive smoking animal poisoning system (PAB-S200, Beijing Bestlab High-Tech Co., Ltd. Beijing, China) composed of 80 (length) × 60 (width) × 58 (height) cm^3^ chamber that is separated into two layers. The smoke generated by commercially filtered cigarettes (0.8 mg of nicotine, 11 mg of tar, and 13 mg of carbon monoxide per cigarette) was directed into the chamber to achieve the condition of passive exposure. The MG rats were daily subjected to two 1-h periods of CSE (morning and afternoon,) for 16 weeks (Fig. 1). The 16th week of CSE was divided into four stages. The amount of cigarette smoke received by rats increased gradually with the progress of smoking time. Throughout the process of smoke exposure, the concentration of carbon monoxide was maintained at 310–380 ppm, while that of oxygen was ≥18%. Two rats died of acute respiratory tract inflammation during the experiment. Thus, only 6 MG rats were evaluated.

### Weight and grip strength

The weight and forelimb grip strength of rats were monitored monthly using a small animal electronic scale (HL-DDC, Beijing Heli Science and Technology Company, Beijing, China) and a small animal grab tester (DM.7-YLS-13A; Dameida Technology Co., Ltd., Beijing, China), respectively. The grip strength tests were performed by placing the rats on the horizontal frame of the instrument and slowly dragging their tails backward so as to suspend their hind limbs. Afterwards, the forelimbs of the rats were forced to grasp the dynamometer level frame, and their tails were pulled back even further, until they were forced to loosen their grip on the level frame. The force required to slacken each rat’s grip was then recorded as the absolute grip strength (AGS). To minimize error, the grip strength tests were repeated five times for each rat in the CG and MG groups, and the average value was ultimately reported. The interval of each test was 30 s. Furthermore, the normalized absolute grip strength (NAGS, NAGS = strength / weight) was calculated to directly control the role of relative mass in muscle strength capabilities.

### Pulmonary function test

The pulmonary function testing system of small animals (Buxco PFT, Data Science International Company, St Paul, USA) was used to analyze the pulmonary function of the investigated rats. Briefly, 24 h after the last CSE session, the CG and MG rats were fasted for 12 h, then they were anesthetized with 10% chloral hydrate (400 mg kg^− 1^ body weight). Ten minutes later, the rats entered the state of deep anesthesia, wherein their righting reflex and pain stimulus response disappeared. No symptoms of peritonitis were detected. After preparation and fixation, the cervical skin of rats was cut longitudinally, and the subcutaneous tissue was bluntly separated. The trachea was then cut and connected with PFT in order to perform the pulmonary function test. Forced vital capacity (FVC), forced expiratory volume in 100 ms (FEV100), forced expiratory volume in 200 ms (FEV200), functional residual capacity (FRC), expiratory reserve volume (ERV), and maximum mid-expiratory flow (MMEF) were measured and used to assess pulmonary function in the investigated rats.

### Muscle strength in vitro

An isolated tissue perfusion system (Automatic organ bath, Panlab Harvard Apparatus, Holliston, USA) was used along with the appropriate data acquisition equipment (Power Lab 4/35, ADInstruments; Oxford, UK) in order to test for muscle strength in vitro. Before testing, a Krebs-Hanseleit buffer (37 °C) was prepared and mixed with a gaseous solution of 95% O_2_ and 5% CO_2_ for 15 min. The two-gram code was used for preload calibration. Under deep anesthesia, the right lower extremity skin of rats was removed, and the tibialis anterior muscle was separated. Afterward, the muscle was placed in a glass bath containing the mixture of Krebs-Hanseleit buffer and O_2_/CO_2_ gas. One end of the muscle was fixed at the bottom of the bath, while the other end was attached to the tension sensor. A tungsten wire electrode (voltage 10 V) was used to stimulate the muscle strips by producing a square electrical wave (frequency = 1 Hz and wavelength = 2 ms) along the vertical axis of the muscle. The maximum peak of muscle contraction was recorded to reflect muscle strength [18].

### Sample preparation

The abdominal cavity of rats was opened under deep anesthesia, and 5 mL of blood were collected from the abdominal aorta. The blood was kept at room temperature, for 30 min, then it was centrifuged at 12000 rpm and 4 °C for 5 min. The supernatant was then separated and analyzed for inflammation levels. The left lung bronchoalveolar lavage fluid (BALF) extracted after tracheal intubation (3 cycles) was used to collect 3 mL saline (70–80% recovery), then it was centrifuged at 3000 rpm for 10 min. Again, the supernatant was separated for the detection of inflammation level. Finally, after pulmonary function test, muscle strength test in vitro and BALF collection, rats were euthanized by decapitation under deep anesthesia. Their right lungs were removed and fixed in 4% paraformaldehyde solution after cardiac arrest and the disappearance of nerve reflex. Part of the gastrocnemius muscle extracted from each rat was also fixed in 4% paraformaldehyde; however, the other part was preserved at − 80 °C for Western blotting analysis.

### Histological staining & morphological analysis

The morphological changes in lung and gastrocnemius tissues were observed by hematoxylin-eosin (HE) staining. After labeling and washing, the tissues were dehydrated, waxed, embedded, and cut into transparent sections using a paraffin microtome (RM2235, Leica, Wetzlar, Germany). Later, the sections were dewaxed, hydrated, then stained with HE. The stained samples were subsequently dried in the air, and their morphologies were observed using a DP80 optical microscope (Olympus, Tokyo, Japan). For the purpose of comparison, three HE - stained sections were randomly selected from each group, and three visual fields were analyzed in each group. The images were recorded and analyzed under 200-fold magnification using the Image-Pro Express system (Media Cybernetics, Maryland, USA). Ten values of alveoli cross-sectional areas (CSA) were calculated determined by the random field of vision, and the average CSA value was calculated and reported. The morphological analyses were also conducted on more than 30 muscle fibers, and on more than three independent CSA in each fiber. The average CSA of muscle fibers was normalized to body weight to exclude the influence of body weight.

### Elisa

The expressions of interleukin (IL)-6, IL-8, and tumor necrosis factor (TNF)-α in serum and BALF were detected by ELISA. After loading and mixing, the sample was incubated at 37 °C for 60 min, then the liquid was discarded. The sample in each well was washed 5 times before adding the enzyme-labeled reagent (no reagent was added to the blank well). The mixtures were subsequently shaken and incubated at 37 °C for 60 min. After cleaning the microplate, the visualization reagent was added and the color was developed at 37 °C for 10 min before adding the stop buffer. Finally, the optical density (OD) of each well was measured, and the corresponding sample concentration was calculated.

### Western blotting

The molecular weights of the Atrogin-1, MuRF1, and GAPDH (internal reference) target proteins are 42, 40, and 36 kDa, respectively. The total protein contents in rats’ gastrocnemius muscles were extracted and determined according to the BCA method. The BCA working fluid and standards used in protein analyses were prepared based on the instructions of the BCA protein concentration determination kit (Beyotime Biotechnology Co., Ltd., Shanghai, China). Briefly, the samples were mixed with buffer solution (5 times their volume) and boiled for 15 min to denature the protein. SDS-PAGE and membrane transfer were carried out, and the membrane was blocked with 5% skimmed milk for 1 h. Thereafter, the diluted first antibody solution was added [rabbit anti-Atrogin-1 antibody (ab74023, Abcam, 1:1000), mouse anti-MuRF1 (sc-398,608, Santa Cruz, 1:1000)], and the mixtures were incubated at 4 °C overnight. Then, the samples were washed before adding the second antibody (5% BSA solution 1:5000) and incubating at room temperature for another 1 h. Finally, a photosensitive solution was added to each sample, resulting in the formation of a photosensitive film that was fixed and scanned. The scanned images were analyzed by ImageJ software, and the optical density of each band was measured. The OD ratios of the target and GADPH bands were used to assess the relative protein expressions.

### Statistical analysis

Changes in the functional indexes (pulmonary and muscle functions) and muscle protein levels of experimental animals are considered to be key indicators of skeletal muscle dysfunction. Inflammation levels and lung and skeletal muscle structures are also important factors. Therefore, these features were numerically assessed, and the corresponding values are expressed as mean ± standard deviation (SD). It should be noted that all data values reported herein were tested for normality and homogeneity of variance. The temporal evolution (1, 2, 3, and 4 months of CSE) of the forelimb grip strengths and body weights of rats were analyzed by two-way repeated-measures ANOVA, whereas independent sample t-tests were used to evaluate the inter-group variations of other indicators. Differences were considered to be statistically significant if *p* < 0.05. The data were processed using the SPSS 23.0 software.

## Results

### General observations and forelimb grip strength

As the CSE experiment progressed, the rats in MG took less food and water, and they behaved more irritably. Moreover, their breaths became short and loud upon smoke exposure, and the color of their furs turned yellowish. Comparatively, the CG rats showed no signs of irritation, and their diet, respiratory rhythm, and fur color (white) remained unchanged.

**Fig. 2:**
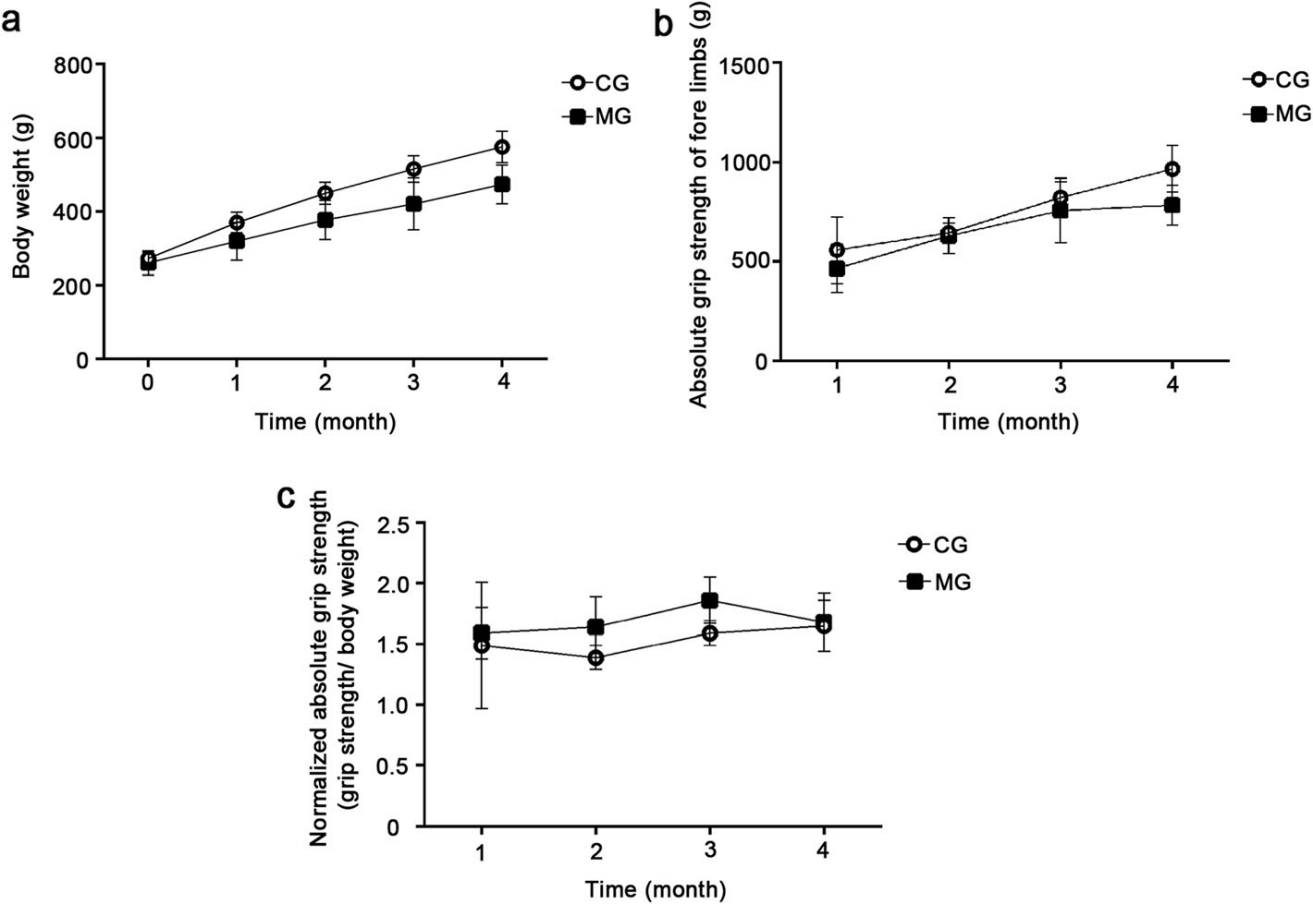
Changes in body weight and fore limb grip strength. **a**: The weight change of rats during the establishment of the model. **b**: The forelimb AGS change of rats during the establishment of the model. **c**: The forelimb NAGS change of rats during the establishment of the model. MG, model group; CG, control group

The baseline levels of body weight were the same in MG and CG, and both groups showed an increase in rat weight during the experiment. However, the weights of MG rats increased more slowly than those of CG rats (Fig. 2a, Group: F = 28.296, *p* = 0.003; Time: F = 173.976, *p* < 0.001; Interaction: F = 9.471, *p* = 0.008). The results illustrated in Fig. 2a indicate that the body weights of rats in the two groups are appreciably affected by the time factor (*p* < 0.001). Comparisons within the same group show that rats exhibit significantly different body weights (*p* < 0.05) after 1 and 2 months of CSE experimentation; however, the variations are reduced on the 3rd and 4th months of exposure (MG: *p* = 0.211, CG: *p* = 0.062). Beyond the 1st month of CSE (F = 4.275, *p* = 0.094), the differences in the averaged body weights of MG and CG rats become significant (*p* < 0.01).

The AGS of rats also increased with time (Fig. 2b, Time: F = 31.929, *p* < 0.01; Group: F = 8.323, *p* = 0.034; Interaction: F = 1.473, *p* = 0.280). However, the rate of increase was found to be much higher during the first 2 months (*p* < 0.05) of modeling than after 3 and 4 months of CSE (*p* > 0.05). Moreover, the grips of MG rats were found to be substantially weaker than those of CG rats (*p* = 0.034). Nevertheless, the 16-week period of model-buliding did not cause significant changes in NAGS between two groups (Fig. 2c, Group: F = 2.435, *P* = 0.179; Time: F = 3.027, *P* = 0.062; Interaction: F = 0.814, *P* = 0.435).

### Pulmonary function and histology

**Fig. 3:**
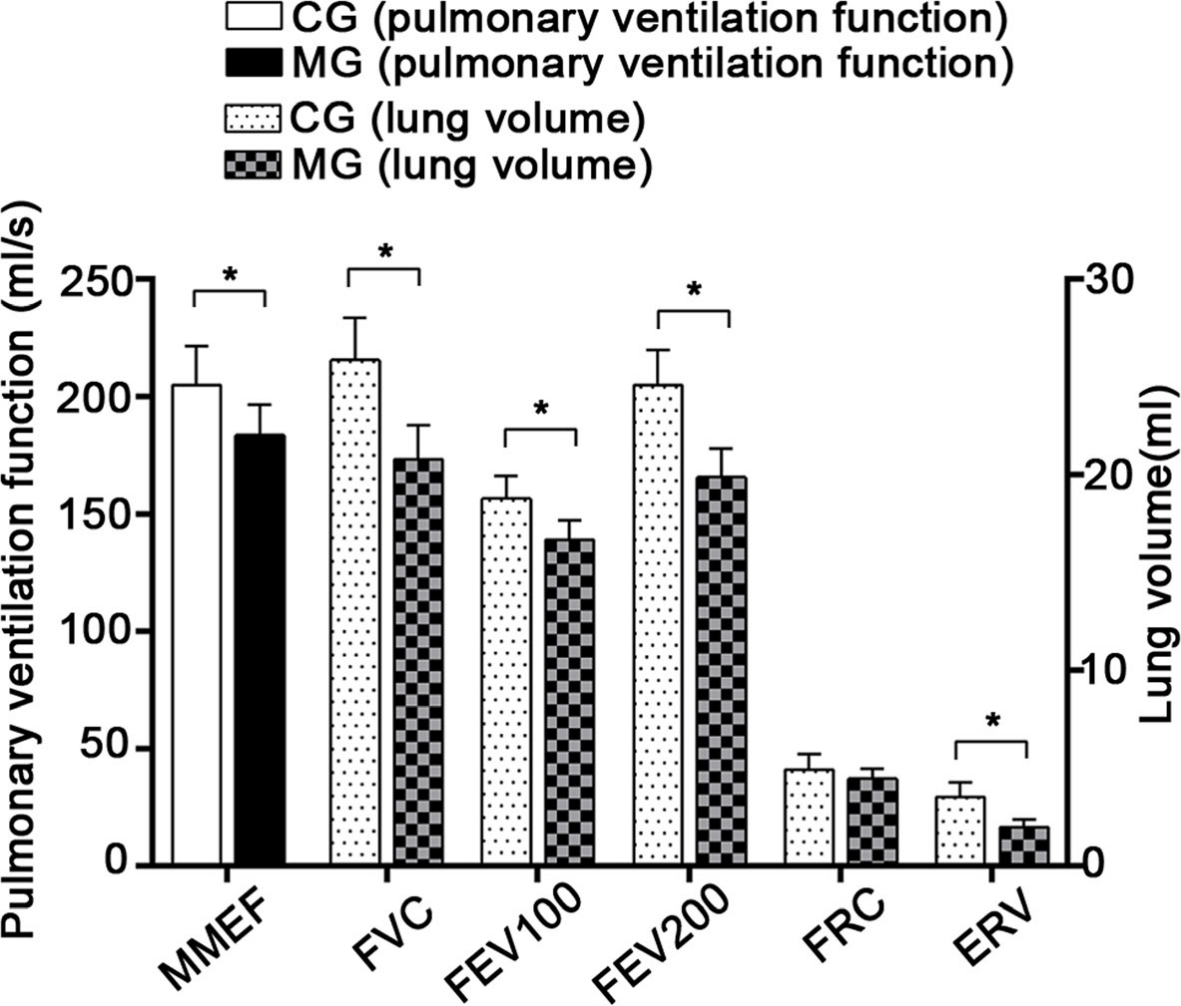
Pulmonary function between two groups. *indicates *p* < 0.05. MMEF, maximal mid-expiratory flow; FVC, forced vital capacity; FEV100, forced expiratory volume in 100 milliseconds; FEV200, forced expiratory volume in 200 milliseconds; FRC, functional residual capacity; ERV, expiratory reserve volume; MG, model group; CG, control group

Once the skeletal muscle dysfunction model was established, variations in lung volume indexes were observed between the two groups. Compared to CG rats, the ERV of MG rats were significantly reduced (*p* < 0.05). However, the difference in FRC was found to be insignificant. As for the lung ventilation parameters (FEV100, FEV200, FVC, and MMEF), they were appreciably lower in model rats than in control rats (*p* < 0.05), which indicates that the CSE inhibits ventilation function of rats (Fig. 3).

**Fig. 4:**
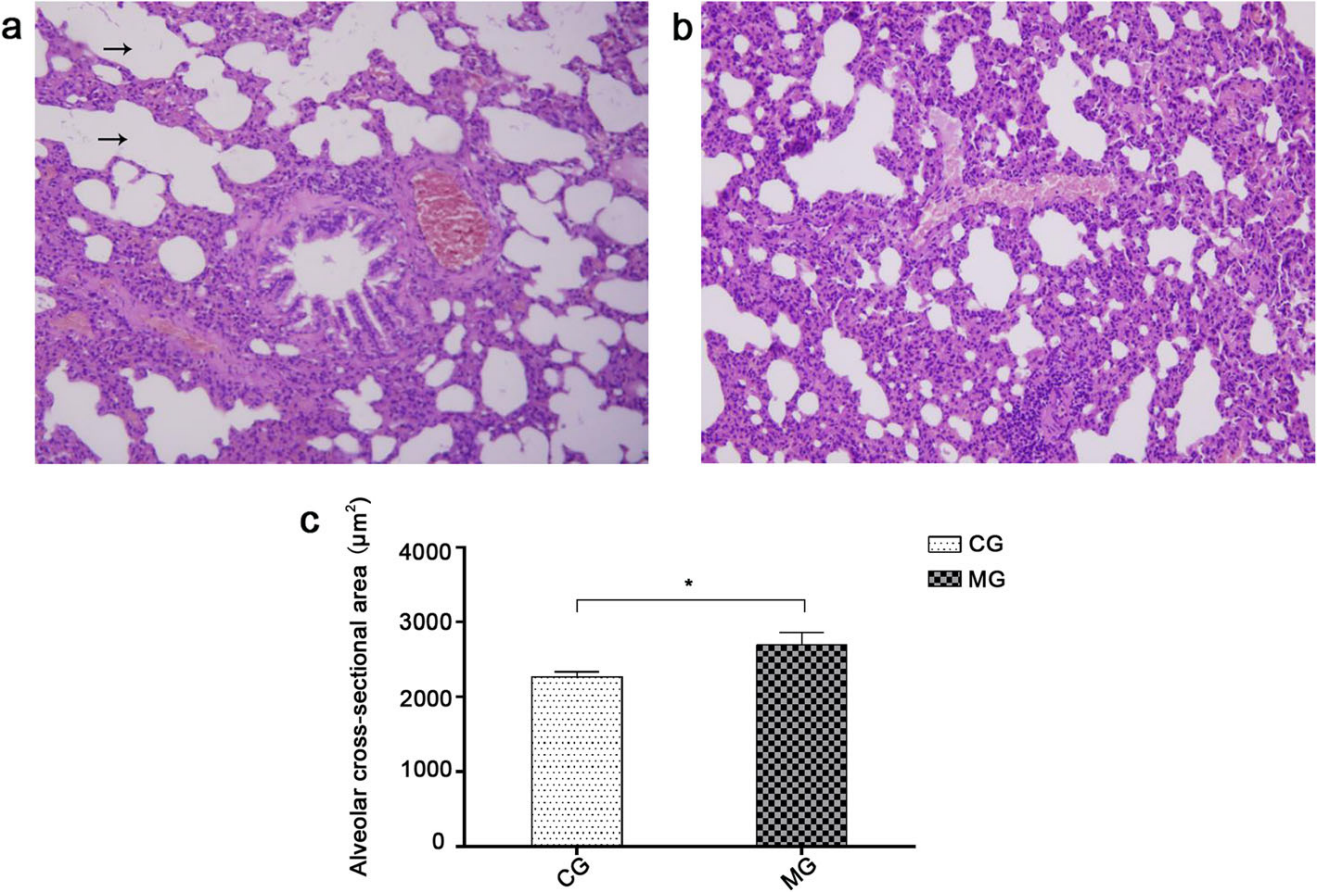
Histological structure of lung. **a**: Histological structure of lung in the model group. The black arrow indicates the enlarged terminal respiratory tract. Magnification: × 200. **b**: Histological structure of lung in the control group. Magnification: × 200. **c**: Comparison of the alveolar cross-sectional area between two groups of rats. *indicates *p* < 0.05. MG, model group; CG, control group

After 4 months of repeated smoke exposure, the rats in MG presented pathological evidence of COPD, including a large amount of interstitial hyperplasia in lung tissue sections, thickened airway walls, columnar ciliated epithelium lodging, deletion, and adhesion, disordered alveolar structure, and increased number and volume of alveolar sacs (Fig. 4a). Meanwhile, the rats in CG showed normal tissue structures of the pulmonary interstitium, tracheal wall, and alveoli (Fig. 4b). Additionally, the alveolar CSA in MG was found to be significantly larger than that in CG (*p* < 0.05; Fig. 4c). Overall, the pathological observations discussed above indicate that MG rats suffer from emphysema.

### Skeletal muscle function and histology

**Fig. 5:**
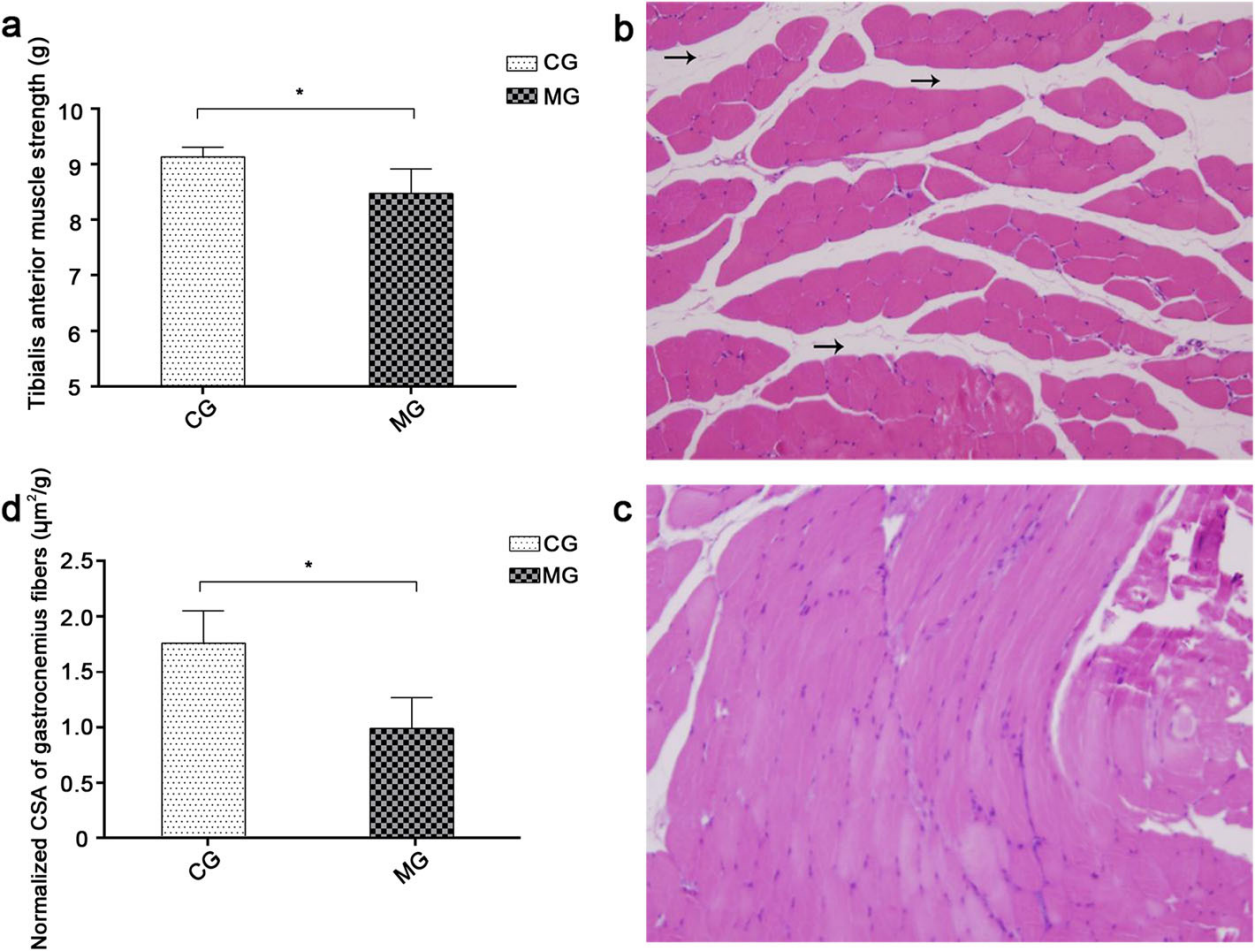
Muscle strength of tibialis anterior muscle in vitro and histological structure of gastrocnemius muscle. **a**: Contractility of tibialis anterior muscle in vitro. *indicates *p* < 0.05. **b**: Histological structure of gastrocnemius in the model group. Black arrows indicate enlarged muscle fiber spaces. Magnification: × 200. **c**: Histological structure of gastrocnemius in the control group. Magnification: × 200. **d**: Comparison of normalized CSA of gastrocnemius muscle fibers between two groups of rats. *indicates *p* < 0.05. CSA, cross-sectional area; MG, model group; CG, control group

The contractility of the tibialis anterior muscle in MG rats decreased throughout the 16-week period of model-building. Compared to CG, MG showed substantially weaker and less contractile tibialis anterior muscles (*p* < 0.05) (Fig. 5a). Moreover, after 4 months of smoke exposure, the fibers of the gastrocnemius muscle became somewhat separated, the septa of muscle fibers became large (abnormal tissues), and the blood vessels and capillaries were more proliferated (Fig. 5b). Comparatively, CG rats showed normal gastrocnemius muscle structure, with tightly and neatly arranged fibers. Moreover, the individual muscle fiber bundles in CG rats were found to be closely packed and unseparated, and no evidence of hyperplasia was detected (Fig. 5c). The normalized CSA of the gastrocnemius muscle fibers in MG rats was found to be appreciably smaller than that estimated for CG rats (*p* < 0.05) (Fig. 5d). These results indicate that long-term CSE may reduce the volume of gastrocnemius muscle fiber bundles, resulting in the onset of dysplasia.

### Inflammation level in rats

**Fig. 6:**
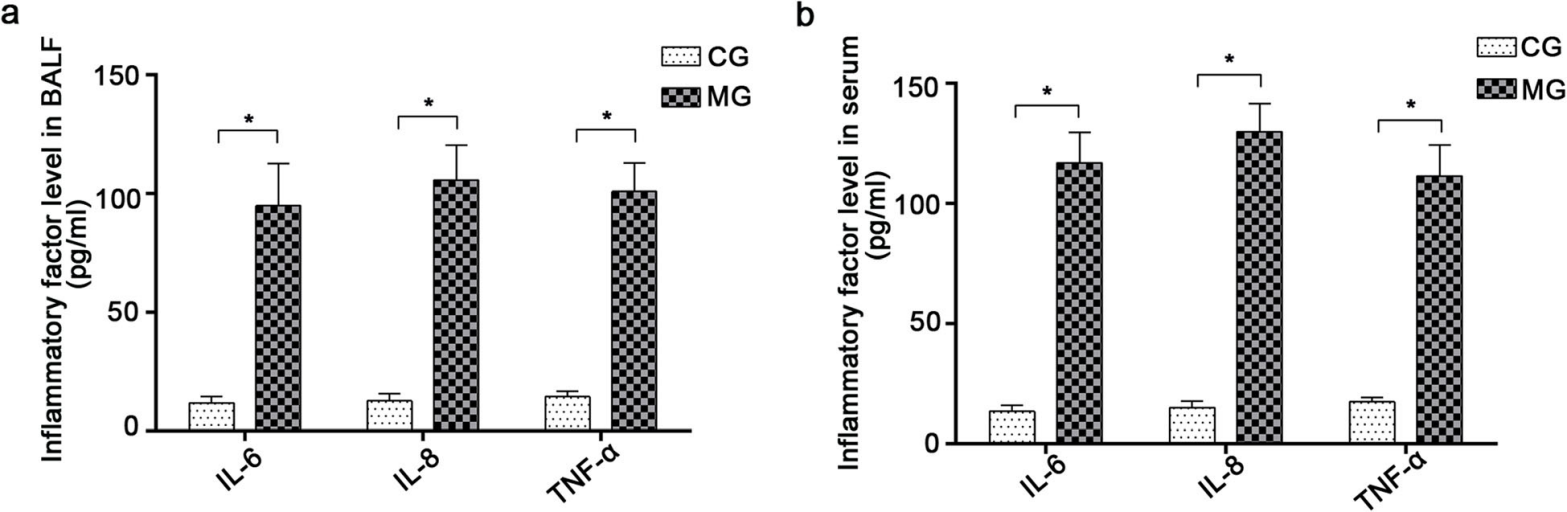
Expression of inflammatory factors in BALF and serum. **a**: Expression of inflammatory factors in BALF. *indicates *p* < 0.05. **b**: Expression of inflammatory factors in serum. *indicates *p* < 0.05. BALF, bronchoalveolar lavage fluid; MG, model group; CG, control group

The expressions of BALF inflammatory factors (IL-6, IL-8, and TNF-α) in MG were found to be significantly higher than those in CG (*p* < 0.05), which suggests that CSE causes lung airway inflammation (Fig. 6a). Similarly, the expressions of IL-6, IL-8, and TNF-α were found to be much higher in MG than in CG (*p* < 0.05). Therefore, in addition to airway inflammation, long-term CSE induces systemic inflammation in rats (Fig. 6b).

### Expressions of Atrogin-1 and MuRF1 in the gastrocnemius muscle

**Fig. 7:**
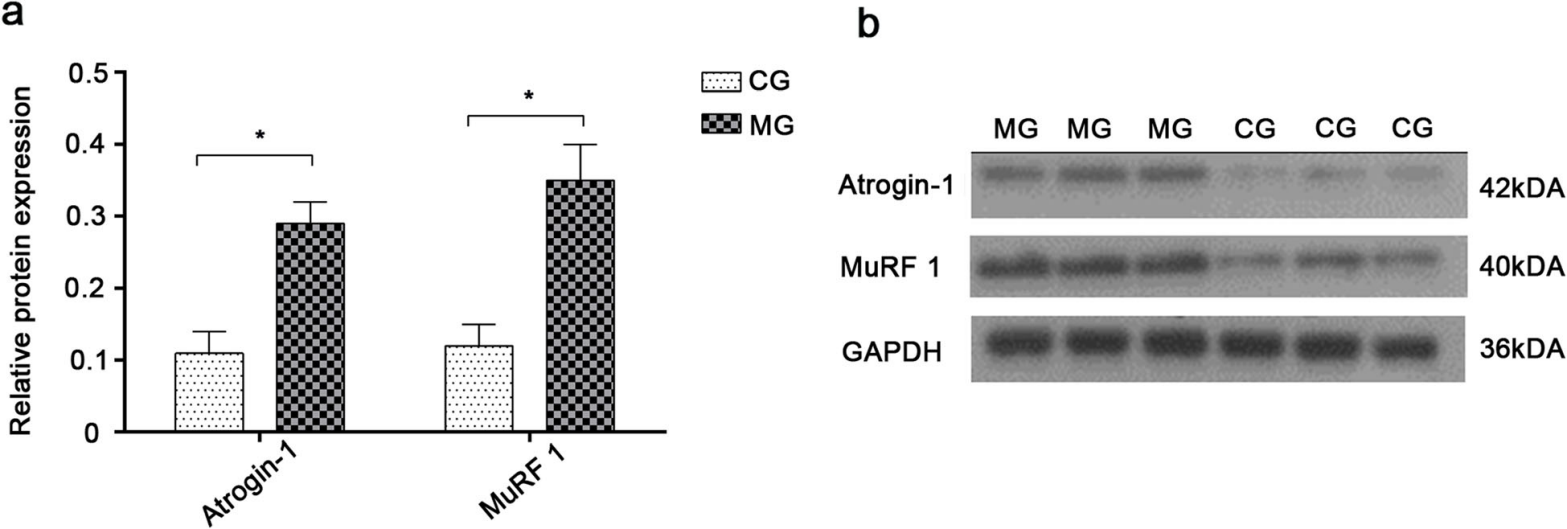
Expression of Atrogin-1 and MuRF1 in gastrocnemius muscle and Western blotting images. **a**: Expression of Atrogin-1 and MuRF1 in gastrocnemius muscle. *indicates *p* < 0.05. **b**: Representative images of immunoblots showed more abundant Atrogin-1 and MuRF1 proteins in COPD gastrocnemius muscle as compared with of normal muscle controls as determined by an immunoblotting assay. CG, control group; MG, model group

Compared with the CG, the expressions of Atrogin-1 and MuRF1 proteins in the gastrocnemius muscle tissue of model rats were shown to be substantially high (*p* < 0.05). This indicates that muscle protein of MG rats is significantly degraded, and that these rats suffer from skeletal muscle dysfunction (Fig. 7).

## Discussion

### Establishment of the COPD skeletal muscle dysfunction model in rats

Skeletal muscle dysfunction is an important factor affecting the prognosis and quality of life of COPD patients. To develop effective treatments for this condition, a mature model needs to be established and researched. Over the past 20 years, a variety of methods have been proposed for the establishment of the COPD model. However, the appropriate method for developing an animal model of COPD skeletal muscle dysfunction remains unclear. This study aims to validate the currently recognized scheme of COPD rat model preparation and to determine whether smoke exposure, could be used to establish the COPD skeletal muscle dysfunction model.

Previously reported studies indicate that the time required to successfully establish a COPD model varies. In general, the model can be quickly set up by combining smoke exposure with intratracheal dripping of pro-inflammatory agents. For example, Song et al. [19] established the COPD model by exposing rats to smoke for 1 month, and injecting them with lipopolysaccharide (LPS) before and during exposure. Their results indicate that the airway BALF inflammatory factors and pulmonary functions of the model rats are significantly different from those of normal rats. In light of its time-effectiveness, this method is still being used by researchers to establish COPD animal models. Another available method is the one proposed by Zhen et al. [20] who established the COPD model in 90 days, simply by exposing rats to Hataman cigarette smoke (two 1-h sessions of smoke exposure, per day) and infusing them with elastase (2 U kg^− 1^ body weight) on the 30th day. The model has also been set up by combining Klebsiella pneumonia dilution with incremental CSE [21].

Although it is possible to develop the COPD model over a short period of time, the duration of COPD formation in patients is long-term, and the initial symptoms are usually not obvious. Moreover, COPD is mainly caused by smoking and air pollution since patients are not likely to have come in contact with pro-inflammatory drugs. To assess the effect of drugs in establishing the COPD model, Nie et al. [17] made a comparison between rats exposed to smoke only and rats subjected to smoke and LPS. Their results show that the former model exhibits pathologically consistent manifestations of COPD, whereas the latter shows acute deterioration. This suggests that although the use of drugs can speed up the process of establishing a COPD model, it stimulates pathogenic pathways that are not involved in the actual human disease. Therefore, in order to approach the conditions of COPD development and progress in patients, many researchers rely on the exclusive use of CSE in model preparation. For example, Zheng et al. [22] developed the COPD model by subjecting rats to 2 h of CSE per day for 252 days. A successful model has also been established via continuous CSE for 16 weeks [23].

The establishment of a COPD skeletal muscle dysfunction model is confirmed by the detection of impaired lung and skeletal muscle functions. Previously, it has been shown that long-term exposure of animals to large amounts of smoke leads to the onset of COPD, as well as other common complications such as weight loss and skeletal muscle dysfunction [24, 25]. In particular, Davidsen et al. [24] reported that after long-term exposure to smoke (3 months, 5 days per week, 4 cigarettes per session), the gene expressions in guinea pigs reflect skeletal muscle atrophy similar to that observed in COPD patients. However, no attempts were made to relate the pathological levels and functions. Caron et al. [26], on the other hand, showed that after 24 weeks of smoke exposure (5 days per week, 12 cigarettes per session) the gastrocnemius and soleus muscles of the model animals become significantly smaller than those of the control animals, which indicates that the duration of smoking is closely related to the development of skeletal muscle dysfunction. Despite its importance, Caron et al.’s study fails to examine the effect of CSE on skeletal muscle function. Overall, the studies available in the literature prove that skeletal muscle dysfunction can be triggered by long-term CSE; therefore, this method is suitable for the preparation of the COPD skeletal muscle dysfunction model in rats.

In this work, we further explore the efficiency of long-term, incremental CSE in establishing a COPD skeletal muscle dysfunction model. We used a similar total number of cigarettes as Caron et al. [26]; however, we shortened the modeling duration to 16 weeks. To confirm the successful establishment of the model, we assessed the functions and histological structures of rat lungs and skeletal muscles. The levels of inflammation and protein degradation in the muscles were also evaluated.

### Effect of incremental smoke exposure on lung function and structure in rats

The reduced function and altered structure of lungs are considered to be important indicators of COPD [20, 27]. In clinical pulmonary function tests, the specific manifestations of COPD p include decreased FEV1/FVC levels [28], as well as reduced expressions of MMEF and other ventilation indicators [29]. Diminished levels of basic and superimposed lung volume indicators, such as ERV, inspiratory reserve volume (IRV), and FRC, are also indicative of COPD [30]. Unlike clinical data, the pulmonary data collected from animal models are affected by existing instruments, and the indicators of different instruments may vary. Moreover, although the use of PFT in this study renders the clinical and animal models somewhat similar, some of the indicators assessed herein are not fully consistent with those evaluated in clinical tests. For example, unlike clinical examinations, FEV100 and FEV200 are used as ventilation indicators to reflect the lung ventilation function in animal studies [31]. Our results show that ERV, FEV100, FEV200, FVC, and MMEF levels decrease significantly after exposure to cigarette smoke, which means that 16 weeks CSE effectively inhibits the ventilation function of lungs in rats.

CSE also has adverse effects on the lung structure of model rats. Clinically, COPD is mainly manifested in the form of emphysema and chronic bronchitis [32]. Pulmonary emphysema models are diagnosed by alveolar rupture, fusion, and an obvious increase in the alveolar CSA. Meanwhile, chronic bronchitis is evidenced by airway inflammation and the infiltration of a large number of inflammatory cells into the lung tissue. These symptoms have been previously observed in COPD rat models [17]. The model established by Zheng et al. [22] presents additional evidence of impaired pulmonary structure, including excessive airway mucus, accumulated inflammatory cells in the lung tissue and thickened layers of epithelial cells. Moreover, the alveolar CSA of model rats was found to be significantly higher than that of normal rats. The results of HE staining analysis performed herein indicate that the MG rats exhibit thickened tracheal wall, narrowed tracheal lumen, fused and ruptured pulmonary alveoli, and increased alveolar CSA. This confirms that 16 weeks of smoke exposure destroys the lung structure of model rats producing pathological changes specific to COPD. The structural changes observed in this study are consistent with the declined pulmonary function discussed earlier, and they both indicate that the COPD model may be successfully established using our method.

### Effect of smoke exposure on the skeletal muscles of rats

Functionally, skeletal muscle dysfunction is characterized by reduced muscular strength and shortened duration of muscular contraction. The assessment of muscle contractility of forelimb indicates that CSE significantly inhibits the active contraction of muscles, manifested as the decrease of AGS of the forelimb. Moreover, CSE slows down the process of weight gain in rats, resulting in the MG rats being lighter than CG. These results agree well with previous reports of weight and muscle strength loss in COPD mice exposed to cigarette smoke [25]. They are also consistent with the observations of Kamiide et al. [33], who show COPD rats exhibit substantially weaker grips than normal rats. However, the 16-week CSE did not affect NAGS in MG rats. The difference between body weight and AGS gain ratio may contribute to this result: the gain ratio of body weight in CG was significantly higher than that of model rats, which brought about smaller NAGS in CG. Moreover, NAGS showed no significant difference between the two groups, indicating that body weight was an essential factor affecting muscle function [34]. Muscle mass may play a key role: the studies [35–37] available in the literature prove that protein synthesis of myocyte is weakened and the muscle growth is inhibited in COPD, which means that the modeling process may inhibit muscle growth and then inhibit muscle function. Although these hypotheses are not reflected in our results, it suggests that CSE may cause skeletal muscle dysfunction by inhibiting muscle growth, taking into account the importance of muscle mass in body weight.

To further analyze the influence of CSE on muscle strength and contractility [38], the tibialis anterior muscle was completely dissected and examined. Such examination allows for the assessment of the effect of CSE on the strength of the sectioned muscular tissue, irrespective of the size and tailoring of the muscle. The obtained results suggest that even after dissection, the model rat muscles are less contractile than those of healthy rats. Such muscular atrophy is attributed to the reduced volume or number of muscle fibers, and it is considered to be one of the important morphological changes that are indicative of skeletal muscle dysfunction [39]. Previously, Basic et al. [40] had shown that long-term smoke exposure produces symptoms of gastrocnemius muscle dysfunction in COPD model rats. These symptoms include muscle fiber atrophy and decreased exercise endurance. The gastrocnemius muscle controls the flexion of the knee joint, as well as the ankle phalanx, and thus, it is an important motor muscle of the lower extremities. As such, it is often selected for the observation of morphological changes associated with skeletal muscle dysfunction. In our study, we observed sparsely distributed muscle fibers in the gastrocnemius of model rats, compared to more closely packed fibers in normal rats. Moreover, the MG rats show interstitial hyperplasia between their muscle fibers, with a disordered and uneven distribution of muscle nuclei. Normalized CSA of muscle fibers was calculated to exclude the effect of body weight on the muscle fibers. The smaller normalized CSA of gastrocnemius muscle fibers in MG suggests that the COPD rats in this group exhibit reduced volumes of skeletal muscle fibers. The reduction of normalized CSA of muscle fibers is indeed vital evidence of muscle atrophy. The functional and morphological changes discussed above are both consistent with the condition of skeletal muscle dysfunction. Therefore, our results show that CSE inhibits the function of fore and hind limb muscles in model rats.

Knowing that muscle dysfunction in COPD patients is directly correlated with the degradation of synthetic muscle fiber proteins by ubiquitin protease [41, 42], the effect of cigarette smoke on myoprotein degradation was also examined. Specifically, we determined the expressions of the Atrogin-1 and MuRF1 E3 ligases responsible for identifying the target proteins destined for degradation [43]. One study available in the literature shows that the expression of Atrogin-1 in muscle cells and tubes of COPD patients is significantly higher than that in healthy individuals [44]. Another study demonstrates that COPD rats showing significantly reduced grip strength and aerobic exercise capacity also exhibit increased levels of E3 ligase proteins in their skeletal muscles [45]. Consistently, our results indicate that the expressions of Atrogin-1 and MuRF1 in the gastrocnemius of COPD rats are higher than those of control rats. Therefore, we may conclude that long-term CSE could lead to muscle protein degradation in COPD rats. Further analyses are needed to elucidate the specific mechanism of muscle protein degradation by CSE; however, the available data suggests that this mechanism is mediated by oxidative stress (increased levels of reactive oxygen species) [46].

### Effect of incremental smoke exposure on the level of inflammation in rats

Inflammation is the core mechanism of COPD and the main factor leading to extrapulmonary effects, such as skeletal muscle dysfunction [47]. The factors commonly associated with inflammatory reaction are IL-6, IL-8, and TNF-α. IL-6 is known to expand the range of inflammation, particularly in the airway sputum, BALF, and serum of COPD patients. Meanwhile, IL-8 has a chemotactic effect on neutrophils and monocytes, and its levels are significantly increased in the sputum and BALF of COPD patients. The expression of this factor is closely related to the aggregation and infiltration of inflammatory cells. As for TNF-α, it can also exacerbate inflammation in the airway sputum and serum of COPD patients. Similar to prior studies [48, 49], our results show that the expression levels of IL-6, IL-8, and TNF-α in the BALF of COPD rats are higher than those in normal rats, which indicates that the model group exhibits airway inflammation. This is confirmed by the detection of increased levels of inflammatory factors in the serum of rats exposed to cigarette smoke. The systemic inflammatory response identified in our study is actually the main phenotype of COPD, and it exists in the lung and surrounding skeletal muscles [50, 51]. The detection of such systemic inflammation further supports the successful establishment of the COPD skeletal muscle dysfunction model. It should be noted that cigarette smoke can induce inflammatory reactions at both, the tissue and cellular levels, leading to skeletal muscle dysfunction and the inhibition of muscle formation. This hypothesis is confirmed by Huang et al. [52] who report that CSE increases the level of TNF-α in the gastrocnemius muscle and C2C12 cells, while decreasing the expressions of histone deacetylase 2 and other factors related to muscle production.

Based on the results of discussed above, it may be concluded that 16-week CSE negatively impacts the morphology and function of skeletal muscles in COPD rats, thereby inducing skeletal muscle dysfunction in the animal models. However, the number of rats tested in this study is smaller than other researches, which may limit the efficiency of comparison and the applicability of the results. Therefore, a larger sample size of rats should be investigated in future studies. In our present study, CSE did not cause significant changes in NAGS, and it deserves to be explored in the follow-up study, considering its vital role in reflecting muscle function. Meanwhile, it is unknown whether the isolated muscle strength normalized by muscle mass was changed significantly after modeling. Also, more indicators of related to changes in molecular level, such as apoptosis and autophagy, should be analyzed, and the expression levels of inflammation factors in skeletal muscle should be further defined. Nevertheless, the results reported herein are of great significance for the development of future studies regarding skeletal muscle dysfunction treatment in COPD patients.

## Conclusion

This study investigates the effect incremental CSE (16 weeks) in triggering the onset of skeletal muscle dysfunction in COPD rats. Assessments of pulmonary function, histological structure, and inflammatory factors indicate that CSE can indeed be used to establish the COPD model. Meanwhile, examinations of skeletal muscle function, morphology and key indicators show that continuous long-term exposure to cigarette smoke eventually leads to the onset of skeletal muscle dysfunction in COPD animal models. Therefore, the long-term incremental CSE method proposed in this study can be used to successfully establish a COPD skeletal muscle dysfunction model in rats, which is of great significance for the study of the implicated pathogenic pathways and intervention mechanisms. Further investigations are needed to assess whether a better model could be established using other kinds of animals, and to determine the influence of CSE on other factors of COPD skeletal muscle dysfunction in rats.

## Data Availability

The datasets used and/or analysed during the current study are available from the corresponding author on reasonable request.

## Abbreviations

COPD: Chronic obstructive pulmonary disease
MG: Model group
CG: Control group
IL: Interleukin
TNF: Tumor necrosis factor
ELISA: Enzyme-linked immunosorbent assay
FEV: Forced expiratory volume
CSE: Cigarette smoke exposure
UPS: Ubiquitin-proteasome system
AGS: Absolute grip strength
NAGS: Normalized absolute grip strength
FVC: Forced vital capacity
FRC: Functional residual capacity
ERV: Expiratory reserve volume
MMEF: Maximum mid-expiratory flow
BALF: Bronchoalveolar lavage fluid
HE: Hematoxylin-eosin
CSA: Cross-sectional areas
OD: Optical density
SD: Standard deviation
LPS: Lipopolysaccharide
